# Muscle dissatisfaction in young adult men

**DOI:** 10.1186/1745-0179-2-6

**Published:** 2006-04-04

**Authors:** Anu Raevuori, Anna Keski-Rahkonen, Cynthia M Bulik, Richard J Rose, Aila Rissanen, Jaakko Kaprio

**Affiliations:** 1 Department of Public Health, University of Helsinki, Finland; 2 Obesity Research Unit, Department of Psychiatry, Helsinki University Central Hospital, Finland; 3 Department of Epidemiology, Columbia University, USA; 4 Department of Psychiatry, University of North Carolina at Chapel Hill, USA; 5 Department of Psychology, Indiana University, Bloomington, Indiana, USA; 6 Department of Mental Health, National Public Health Institute, Helsinki, Finland

## Abstract

**Backround:**

Appearance concerns are of increasing importance in young men's lives. We investigated whether muscle dissatisfaction is associated with psychological symptoms, dietary supplement or anabolic steroid use, or physical activity in young men.

**Methods:**

As a part of a questionnaire assessment of health-related behaviors in the population-based FinnTwin16 study, we assessed factors associated with muscle dissatisfaction in 1245 men aged 22–27 using logistic regression models.

**Results:**

Of men, 30% experienced high muscle dissatisfaction, while 12% used supplements/steroids. Of highly muscle-dissatisfied men, 21.5% used supplements/steroids. Mean body mass index, waist circumference, or leisure aerobic activity index did not differ between individuals with high/low muscle dissatisfaction. Muscle dissatisfaction was significantly associated with a psychological and psychosomatic problems, alcohol and drug use, lower height satisfaction, sedentary lifestyle, poor subjective physical fitness, and lower life satisfaction.

**Conclusion:**

Muscle dissatisfaction and supplement/steroid use are relatively common, and are associated with psychological distress and markers of sedentary lifestyle.

## Background

Men are increasingly concerned about the way they look: a moderately or extremely muscular body is widely accepted as an ideal body shape for young men, creating discrepancy between the actual and desired body size and shape [1-5].

Young men who are dissatisfied with their body shape and musculature may be more likely to turn to bodybuilding, dietary supplements, and anabolic steroids to shape their bodies [6,7]. The pursuit of muscularity has been associated with significant behavioural and psychological problems [4]. However, it is also plausible that exercise, weight training, and even competitive bodybuilding may actually improve men's body image [8-10].

Most of the existing studies in this area have focused on defined groups of young men, such as athletes and college students: it is unclear how generalizable their results are. Very little is known about the association of muscle dissatisfaction with physical activity, dietary supplement or anabolic steroid use, or psychological symptoms in young men from the general population. The aim of this study was to describe the psychological and behavioral correlates of muscle dissatisfaction in a population study of young Finnish males.

## Methods

### Sample

The data reported are from a population-based twin study, FinnTwin16, and comprised 1245 adult male twins from the 1977 to 1979 birth cohorts, for whom a questionnaire included items on muscle dissatisfaction (MD) and supplement and steroid use as described below. Data collection was approved by the Ethics committees of the University of Helsinki and Indiana University. The present study is based on the fourth wave of the study (response rate 83%) that assessed mental and physical health, weight control, dietary and exercise habits, social relationships, and alcohol and drug use. Mean age at response was 23.8 y (SD 23.7–23.9), mean BMI was 23.9 (SD 3.1), mean waist circumference 85.3 cm (SD 9.4) and mean height 179.4 cm (SD 6.6). A total of 70% of men studied were at normal body weight (BMI 20.0–24.9), while 26% were overweight (BMI 25.0–29.9) and 4% were obese (BMI 30.0–34.9). Of the study subjects 30% of men were 180–184 cm tall, 28% were 175–179 cm, 17% were 170–174 cm, 15% were 185–189 cm, and 6% were 190 cm or more while 4% were 169 cm or less. At the time of the study, 53% of men were not studying, while 21% attended a college or university, 18% attended a polytechnic, 4% attended high school, vocational school or vocational college and 4% studied in some other school not mentioned above. By this age, 5% of men had reached a higher academic degree while 8% had a degree from polytechnic and 42% from a vocational school.

## Measures

### Muscle dissatisfaction

Muscle dissatisfaction (MD) was assessed based on the following questionnaire item: "I would like to be more muscular". The six original responses were recategorized into "Always or Commonly" (High MD), "Often" (Intermediate MD), and "Sometimes, Rarely, or Never" (Low MD).

### Supplement use

Supplement and/or steroid use (from here on, referred to as supplement use) was assessed based on a question: "Have you ever used hormone preparations, dietary supplements, or other special preparations in order to increase your muscle mass or to maximise the effects of gym training?". The answering alternatives were: "Yes, continuously during the last three months", "Yes, continuously at least three months sometimes earlier", "I have sometimes tried", and "I have never tried nor used". To differentiate established use, supplement use was dichotomized: men who had engaged in supplement use for ≥3 months either recently or previously were considered supplement users, while others were not considered as users.

### Height satisfaction

Height satisfaction was assessed based on a following questionnaire item: "I am satisfied with my height". The answering alternatives were: "Always", "Commonly", "Often", "Sometimes", "Rarely", or "Never".

### Anthropometrics

Values for height (cm), waist circumference (cm) and weight (kg) were based on self-reported data, from which body mass indices (BMI) were calculated. The correlations between self-reported and measured values were excellent, >0.9 for height and BMI, and 0.84 for waist circumference in a subsample of these data [11,12].

### Eating disorder inventory

The questionnaire included the original items of three subscales of the Eating Disorder Inventory-1 (EDI) [13,14]: Body dissatisfaction (BD, 9 items), Drive for thinness (DT, 7 items), and Bulimia (B, 7 items). A Finnish version of this instrument has been translated and validated (Charpentier, in preparation). Scoring for EDI used a range of 1–6 for responses in the questionnaire. DT and B were reasonably normally distributed and were used as continuous variables. However, the distribution of BD was highly skewed, and therefore categorized based on tertiles of the distribution.

### Physical activity and fitness

The participants were asked to characterize their subjective physical fitness using the alternatives: "very good", "fairly good", "satisfactory", "fairly poor" and "very poor". For the analyses, the variable was recategorized into three classes: "Fairly/very good", "satisfactory", "fairly/very poor".

Leisure aerobic activity index (Metabolic Equivalent, MET h × d -1, work metabolic rate divided by resting metabolic rate) was calculated from the product of self reported intensity × duration × days/year frequency. We used MET-values 4 (light aerobic activity, walking), 6 (moderate aerobic activity, brisk walking), 10 (intense aerobic activity, jogging), 13 (very intense activity, running).

We also asked for daily time spent on sedentary leisure interests, for example driving, TV viewing, computer game and Internet use.

### Illicit drug use

Drug use was assessed based on answers to a question "Have you ever used hash, marijuana or other drugs, or have you for example sniffed glue?". The response alternatives were: "Never", "1–3 times", "4–9 times", "10–19 times", "Over 20 times". For the analyses, the variable was recategorized into three classes: "Never used", "Experimental or mild drug use (1–19 trials)", and "Established drug use (over 20 trials)".

### Other psychological measures

The General Health Questionnaire (GHQ-12) is a screening instrument designed to detect psychiatric disorders in community settings and non-psychiatric clinical settings [15]. It has been validated for the Finnish population [16,17]. Rutgers Alcohol Problem Index (RAPI) is a 23-item, unidimensional self-administered screening tool for assessing adolescent problem drinking [18]. Psychosomatic symptom frequencies were assessed based on answers to six items: stomach aches, tension and nervousness, difficulties in falling asleep or nightly awakenings, headache, lower back pain, and pain in the neck and shoulder area [19]. Each item contained four response alternatives which were: "Rarely or never", "About once a month", "About once a week, and "Almost every day".

The questionnaire also included the Life Satisfaction Scale, which covers four items: interest in life, happiness, general ease of living, and loneliness [20]. The scale correlates highly with the Beck depression inventory (r > 0.6) [21].

### Sociodemographic factors

We assessed place of residence (rural vs. urban) using five response categories based on population density. Rural areas were considered to include both rural villages and countryside.

Because the men studied were relatively young and many were still studying, we assessed current educational level in addition to highest educational attainment. The question asked was: "Do you attend a school or college at the moment?" The response alternatives were "I do not attend a school at the moment", "I attend a high school, vocational school or in vocational college", "I attend a polytechnic", "I attend a college or university" and "Other". About educational attainment we asked: "Which schools/degrees have you completed?", using similar response categories as in the previous question.

### Analysis

We investigated associations between independent and dependent variables using cross-tabulations, Pearson's chi-squared test of independence and logistic regression. For continuous variables, odds ratios were calculated for both a unit increase in the dependent variable, and by using a median split to form a dichotomized variable. All analyses were controlled for clustered sampling [22] within the twin pair using Stata (Version 8.0) [23].

## Results

### Characteristics of study sample by muscle dissatisfaction status

Of all respondents, 29.9% experienced high MD, 14.3% intermediate MD, and 55.8% low MD (see Table 1). The subjects of our study were relatively similar in age, height, BMI and waist circumference irrespective of their MD and supplement use status (Table 1). BMI and waist were correlated at r = 0.79 among low MD subjects, r = 0.80 among intermediate MD subjects and r = 0.75 among high MD subjects.

**Table 1:** Characteristics of the study sample by muscle dissatisfaction (MD) status. Means and 95% CI are given for continuous traits

	High MD	Intermediate MD	Low MD
n(% of population)	372 (29.9%)	178 (14.3%)	695 (55.8%)
SU, n (Proportion of MD subgroup subjects)	80 (21.5%)	20 (11.2%)	50 (7.2%)
Mean age, years	23.8 (23.8–23.9)	23.9 (23.8–23.9)	23.8 (23.8–23.9)
Mean BMI, kg/m^2^	23.5 (23.2–23.8)	23.7. (23.2–24.2)	23.9 (23.6–24.1)
Waist circumference, cm	84.1 (83.2–85.1)	85.0 (83.4–86.5)	84.5 (83.7–85.2)
Mean height, cm	180.0 (179.2–180.7)	180.4 (179.3–181.5)	179.0 (178.4–179.5)

Note: No statistically differences between high, intermediate and low muscle disatisfied participants for any traits

### Height satisfaction

Of respondents, 52.5% reported being always satisfied with their height as 28.5% reported this commonly and 8.4% often. A total of 4.7% of men were height satisfied only sometimes, 3.5% rarely and 2.4% never.

Height satisfaction decreased significantly from low to high MD even when adjusted with actual height (OR for always versus commonly 1.6, 95% CI 1.3–2.1, OR for always versus often 1.9, 95% CI 1.4–2.7, OR for always versus sometimes 2.9, 95% CI 1.8–4.8, OR for always versus rarely 3.9, 95% CI 2.3–6.9 and OR for always versus never height satisfied 3.2, 95% CI 1.4–7.6). Those whose actual height was 174 cm or less were significantly more often height dissatisfied (OR 3.9, 95% CI 2.9–5.2), but no more dissatisfied with their musculature (*x*^2 ^= 1.78, p = 0.41) than their taller peers.

### Sociodemographic correlates of muscle dissatisfaction

The place of residence was not associated with MD, but living in a rural area (rural village or countryside) was associated with less supplement use (OR 0.4, 95% CI 0.2–0.8). Current school attendance or a level of educational attainment at this age were not significantly associated with MD.

### Psychological correlates of muscle dissatisfaction

The basic psychometric characteristics of MD subgroup subjects are given in Table 2. The correlates of MD in the univariate models are presented in Table 5. Among young men from the general population, psychological distress as measured by GHQ, psychosomatic symptoms, life satisfaction, drive for thinness and body dissatisfaction were statistically significantly associated with muscle dissatisfaction. These findings were independent of the place of residence and current educational status. Body Dissatisfaction had the strongest association with MD of the three EDI subscales. Eating Disorder Inventory's Drive for thinness and Bulimia were also significantly associated with MD. All three EDI subscales remained significantly associated with MD, even when controlling in regression models for measures of depressive symptoms, i.e. GHQ or life satisfaction.

**Table 2:** Basic psychological characteristics of high/intermediate/low muscle dissatisfied (MD) men: mean scores and SDs.

	high MD	Intermediate MD	Low MD
n	372	178	695
Body dissatisfaction^1^	22.2 (6.4)	21.1 (6.4)	18.6 (5.4)
Drive for thinness^1^	17.4 (4.5)	16.9 (4.3)	16.2 (4.1)
Bulimia^1^	10.2 (2.6)	10.0 (3.0)	9.5 (2.5)
RAPI^2^	30.9 (9.8)	29.0 (8.5)	28.2 (8.1)
Life satisfaction^3^	9.2 (3.2)	9.1 (3.0)	8.1 (2.8)
Psychosomatic symptom score	10.2 (3.1)	9.9 (2.9)	9.2 (2.8)
General Health Questionnaire^4^	2.0 (2.9)	1.7 (2.5)	1.0 (1.9)

^1 ^Eating Disorder Inventory[13]

^2 ^Rutgers Alcohol Problem Index[18]

^3 ^higher score → lower life satisfaction

^4 ^higher score → poorer mental health

### Physical activity and fitness

Higher leisure aerobic activity was not statistically significantly associated with MD (OR 1.0, 95% CI 0.99–1.04) even though the leisure aerobic activity increased categorically with muscle dissatisfaction (Table 4). Instead, MD was associated with longer daily duration (>2 hours/day) of sedentary leisure interests like reading, Internet and TV viewing (OR 1.9, 95% CI 1.12–3.33). MD was also associated with poorer subjective physical fitness, the OR for satisfactory versus good/very good physical fitness being 1.1 (95% CI 0.8–1.3), and the OR for poor/very poor versus good/very good physical fitness being 2.5 (95% CI 1.7–3.9).

**Table 3:** Drug use and supplement use of high/intermediate/low muscle dissatisfied (MD) men based on categoric measures.

	High MD	Intermediate MD	Low MD
Drug use (n, % of MD subgroup)			
Never	258 (69.4%)	122 (68.5%)	524 (75.4%)
Experimental or mild use^1^	82 (22.0%)	46 (25.8%)	130 (18.7%)
Established use^2^	32 (8.6%)	9 (5.1%)	40 (5.8%)
Supplement Use (n, % of MD subgroup)			
SU	80 (21.5%)	20 (11.2%)	50 (7.2%)
Non-SU	292 (78.5%)	158 (88.8%)	645 (92.8%)

^1 ^1–19 trials

^2 ^20 or more trials

**Table 4:** Self-reported leisure aerobic activity and subjective physical fitness of high/intermediate/low muscle dissatisfied men

	High MD	Intermediate MD	Low MD
Leisure aerobic activity (MET) mean value (95% CI)	5.3 (4.7–5.9)	5.2 (4.4–5.9)	4.9 (4.5–5.3)
Subjective physical fitness n (% of MD subgroup)			
Fairly/very good	234 (62.9%)	109 (61.2%)	474 (68.2%)
Satisfactory	96 (25.8%)	52 (29.2%)	189 (27.2%)
Fairly/very bad	38 (10.2%)	14 (7.9%)	26 (3.7%)

**Table 5:** Psychological correlates of muscle dissatisfaction from univariate models. Odds ratios were calculated both per unit increase and on median split (high vs. low) increase.

	OR (per unit)	95% CI	OR, high vs low	95% CI	p-value
*General psychological measures*					
GHQ^1^	1.18	1.12–1.23	-	-	<0.0001
Psychosomatic symptom score^1^	1.11	1.07–1.16	2.65	1.89–3.72	<0.0001
Life satisfaction score^1^	1.12	1.08–1.17	2.55	1.90–3.44	<0.0001
*Substance use and abuse*					
RAPI^1,2^	1.03	1.02–1.04	2.26	1.59–3.22	<0.0001
Drug use					
Never	1.0 (reference)				
Experimental or mild (1–19 trials)	1.28	0.99–1.66	-	-	0.06
Established use (≥20 trials)	1.51	0.95–2.39			0.08
*Eating disorder inventory*					
Drive for thinness^1^	1.06	1.03–1.09	2.60	1.70–3.95	<0.0001
Bulimia^1^	1.09	1.04–1.13	2.09	1.41–3.08	0.0002
Body dissatisfaction					
1^st ^tertile	1.0 (reference)				
2nd tertile	1.99	1.46–2.68	-	-	<0.0001
3rd tertile	3.89	2.94–5.16			<0.0001

^1 ^Dichotomy based on median split

^2 ^Rutgers Alcohol Problem Index[18]

### Substance and alcohol abuse and supplement use

Association with MD and alcohol use measured by RAPI was highly significant (Table 5) (p < 0.0001). Association with MD and drug use was marginally significant, with the OR for never used nor tried versus experimental or mild use being 1.3 (95% CI 0.99–1.66) and OR for never used nor tried versus established use being 1.5 (95% CI 0.95–2.39), see Table 5.

The proportion of supplement users was 12.0% of all respondents: supplement use increased monotonically with muscle dissatisfaction (Table 3). Of non-supplement users, 26.7% experienced high MD compared to 53.0% of supplement users (*x*^2 ^= 47.0, p < 0.001).

### Multivariable analyses of muscle dissatisfaction

In a multivariable model where the same variables as in the univariate model excluding the EDI subscale of Body Dissatisfaction were entered, GHQ score (OR 1.09, 95% CI 1.02–1.16), life satisfaction score (OR 1.65, 95% CI 1.15–2.39), and EDI Drive for thinness (OR 2.05, 95% CI 1.21–3.47) remained statistically significantly associated with MD (Table 6). Psychosomatic symptom score remained marginally significantly associated with MD in a multivariate model (OR 1.5, 95% CI 0.99–2.21).

**Table 6:** Correlates of muscle dissatisfaction from multivariate models. Variables significantly associated with muscle dissatisfaction in univariate models excluding Eating Disorder Inventory's subscale Body Dissatisfaction were entered in the multivariable model. Variables presented in this table were adjusted for all other variables in the model.

	OR	95% CI	p value
*General psychological measures *GHQ score	1.09	1.02–1.16	0.006
Psychosomatic symptom score^1^	1.48	0.99–2.21	0.06
Life Satisfaction Score^1^	1.65	1.15–1.2.39	0.007
*Substance use and abuse*			
RAPI^1,2^	1.33	0.86–2.08	0.20
Drug use			
Never	1.0 (reference)	-	-
Experimental or mild use (1–19 trials)	1.01	0.77–1.33	0.93
Established use (≥20 trials)	0.98	0.57–1.69	0.94
*Eating disorder inventory*			
EDI subscales			
Drive for thinness^1^	2.05	1.21–3.47	0.007
Bulimia^1^	1.03	0.65–1.64	0.89

^1 ^These explanatory variables in the multivariable model were dichotomised using a median split.

^2 ^Rutgers Alcohol Problem Index[18]

## Discussion

Muscle dissatisfaction was strongly associated with psychological distress such as depression and anxiety symptoms, as was hypothesized based on previous studies on body dissatisfaction and related disorders among both men and women [2,4,24]. In our study, lower general life satisfaction, psychosomatic symptoms, lower subjective physical fitness, sedentary lifestyles, lower height satisfaction and problems with alcohol and illicit drug use were also associated with muscle dissatisfaction. In addition, the correlation between muscle dissatisfaction and supplement use was strong. Compared to others, muscle dissatisfied men scored higher on all three EDI subscales referring to potential eating problems and dissatisfaction with one's body shape and weight.

In our current Western culture, appearance concerns are ubiquitous, particularly among young people [25,26]. It is generally accepted that popular culture, advertisement and even toys do not only reflect, but also foster appearance concerns. Very little is known about how these concerns manifest in young men. Our study demonstrates that male appearance concerns are potential indicators of other health problems, such as depressive and anxiety symptoms, and substance use and sedentary lifestyles.

In this population based study we wanted to focus on muscle dissatisfaction since the current young males' body ideal is perhaps primarily based on muscularity. Nevertheless, we do not wish to belittle other aspects of males' body dissatisfaction such as weight and body shape concerns and welcome more sophisticated study designs in the future. In our sample, those more dissatisfied with their musculature were also more dissatisfied with their height even though actual height did not differ between the groups and was adjusted in the regression model. This further signals how high dissatisfaction with one's body easily has more than a single dimension and thus perhaps merely reflects the overall discontent with the self. We also analysed whether relative shortness (height 174 cm or less; 21% of the sample) would be associated with muscle dissatisfaction, but that was not the case.

Muscle dissatisfaction does not appear to be more common among young men who exercise frequently than those who exercise little. There is previous evidence that even demanding training and fitness regimes are not associated with poor body image [8,27]. Rather, muscle dissatisfaction seems to be common in men who are also dissatisfied with many other aspects of their lives, and also tend to rate their subjective physical fitness less favorably than other men. There is evidence that regular exercise is associated with positive psychological health [28,29] and body satisfaction especially in males [30]. Although our study is not able to establish causality, future studies should address whether exercise interventions may also have positive effects on muscle dissatisfaction.

This study explored correlates of muscle dissatisfaction among men in the largest population sample yet reported. The strengths of our sample include its relatively large size, good population coverage, and high response rate. The battery we used was fairly extensive, and included many standardised and validated questionnaires.

Limitations of this study include self-reporting bias: however, an inherent bias in studies of this type. We also measured muscle dissatisfaction using only a single item. Although this item has not undergone extensive psychometric validation, the psychological associations of muscle dissatisfaction were robust and not sensitive to choice of cut-off points. Muscle dissatisfaction was highly correlated with the Body for Dissatisfaction subscale of the EDI, and is probably more appropriate for the measurement of body dissatisfaction in young men, because the EDI mainly focuses on female-specific areas of appearance concerns. Conceptually, muscle dissatisfaction can be regarded to be part of body dissatisfaction, which was therefore not entered into multivariate model like other variables significantly associated with the muscle dissatisfaction in the univariate model. We regret that for practical reasons, the inclusion of longer and more comprehensive measurement of male-specific appearance concerns, like the Muscle Appearance Satisfaction Scale [31], Swansea Muscularity Attitudes Questionnaire [32] or the Yale Brown Obsessive Compulsive Scale Modified for Body Dysmorphic Disorder (BDD-YBOCS) [33] was not an option in this large population study: we highly recommend assessing their use in future studies.

Further limitations of our study include that we had little information about our participants' body composition. By itself, BMI cannot separate lean and total fat body mass, but including waist circumference to these measures was an attempt to compensate for this limitation. Also, the question concerning supplement use did not separate the illegal use of anabolic steroids, other hormones and medicine-like substances from more widely used and legal substances like amino acids or creatinine. However, there is some evidence that creatinine and other nutritional supplements marketed as muscle mass enhancers in Finland have contained small amounts of anabolic steroids [34]. The same phenomenon has been noted internationally [35]. Thus supplement users would not always be aware of the true nature and actual content of the muscle enhancers of their choice.

Finally, we used a large twin sample to address muscle dissatisfaction in the general population. Although twins are in many respects similar to singletons [36] in particular for most psychiatric disorders [37,38], some previous studies suggest that adolescent twins may also be more physically active and physically fit than non-twins [39,40]. It is not known whether these small differences persist into adulthood. Further, twins may differ from non-twins in body size, which in childhood and adolescence is on average slightly smaller compared to singletons. Pietiläinen et al. [41] compared mean body height and mean body weight in the twin cohort used in this study (FT16) when the participants were aged 16–17 y to that of Finnish singletons at the same age and found that by the age of 17 y, the twin boys had reached the height of singleton boys, but still had lower BMIs than the 16.5 y singletons. Our sample suggests that this difference also disappears by young adulthood [14].

## Conclusion

In summary, in young men, muscle dissatisfaction is a broad indicator of poor psychological well-being. It is associated with psychological symptoms and substance use. It was also associated with poor self-rated physical fitness and sedentary lifestyles. Future studies should assess prospectively factors that can improve young men's body image and muscle satisfaction and thus mitigate their psychological suffering.

## Authors' contributions

ARa, AKR, ARi, JK, and RJR participated in the design of the study.

ARa, AKR, and JK carried out the statistical analyses and interpretation of the data.

ARa, AKR, JK, and CMB carried out the drafting of the manuscript

ARa, AKR, CMB, RJR, ARi, and JK carried out the critical revision of the manuscript for the important intellectual content.

ARa, AKR, ARi, and JK obtained funding for the study.

All authors read and approved the final manuscript.

## References

[B1] Epel ES Spanakos A Kasl-Godley J Brownell KD Body shape ideals across gender, sexual orientation, socioeconomic status, race, and age in personal advertisements Int J Eat Disord 1996 19 265 273 10.1002/(SICI)1098-108X(199604)19:3<265::AID-EAT5>3.0.CO;2-K 8704725

[B2] Pope HG Jr Gruber AJ Mangweth B Bureau B de Col Jouvent R Hudson JI Body image Perception among men in three countries Am J Psychiatry 2000 157 1297 1301 10.1176/appi.ajp.157.8.1297 10910794

[B3] Bulik CM Wade TD Heath AC Martin NG Stunkard AJ Eaves LJ Relating body mass index to figural stimuli: population-based normative data for Caucasians Int J Obes Relat Metab Disord 2001 25 1517 1524 10.1038/sj.ijo.0801742 11673775

[B4] McCabe MP Ricciardelli LA Body image dissatisfaction among males across the lifespan: a review of past literature J Psychosom Res 2004 56 675 685 10.1016/S0022-3999(03)00129-6 15193964

[B5] Cohane GH Harrison PG Jr Body image in boys: A review of the literature Int J Eat Disord 2001 29 373 379 10.1002/eat.1033 11285574

[B6] Brower KJ Blow FC Hill EM Risk factors for anabolic-androgenic steroid use in men J Psychiatr Res 1994 28 369 380 10.1016/0022-3956(94)90019-1 7877116

[B7] Field AE Austin SB Camargo CA Jr Taylor CB Striegel-Moore RH Loud KJ Colditz GA Exposure to the mass media, body shape concerns, and use of supplements to improve weight and shape among male and female adolescents Pediatrics 2005 116 e214 220 10.1542/peds.2004-2022 16061574

[B8] Pickett TC Lewis RJ Cash TF Men, muscles, and body image: comparisons of competitive bodybuilders, weight trainers, and athletically active controls Br J Sports Med 2005 39 217 222 10.1136/bjsm.2004.012013 15793091 PMC1725169

[B9] Hausenblas HA Fallon EA Relationship among Body Image, exercise behavior, and exercise dependence symptoms Int J Eat Disord 2002 32 179 185 10.1002/eat.10071 12210660

[B10] Boroughs M Thompson K Exercise status and sexual orientation as moderators of body image disturbance and eating disorders in males Int J Eat Disord 2002 31 307 311 10.1002/eat.10031 11920992

[B11] Silventoinen K Sammalisto S Perola M Boomsma DI Cornes BK Davis C Dunkel L deLange M Harris JR Hjelmborg JVB Luciano M Martin NG Mortensen J Nistico L Pedersen NL Skytthe A Spector TD Stazi MA Willemsen G Kaprio J Heritability of Adult Body Height: A Comparative Study of Twin Cohorts in Eight Countries Twin Res 2003 6 399 408 10.1375/136905203770326402 14624724

[B12] Schousboe K Willemsen G Kyvik KO Mortensen J Boomsma DI Cornes BK Davis CJ Fagnani C Hjelmborg J Kaprio J deLange M Luciano M Martin NG Pedersen N Pietiläinen KH Rissanen A Saarni S Sorensen TIA van Baal GCM Harris JR Sex Differencies in Heritability of BMI: A Comparative Study of Results from Twin Studies in Eight Countries Twin Res 2003 6 409 421 10.1375/136905203770326411 14624725

[B13] Garner DM Eating disorder inventory 2 1991, professional manual Odessa, FL: Psychological Assessment Resources

[B14] Keski-Rahkonen A Bulik CM Neale B Rissanen A Kaprio J Rose RJ Body dissatisfaction and drive for thinness in Finnish young adult twins Int J Eat Disord 2005 37 188 199 10.1002/eat.20138 15822080

[B15] Goldberg DP Hillier VF A scaled version of the General Health Questionnaire Psychol Med 1979 9 139 145 424481 10.1017/s0033291700021644

[B16] Holi MM Marttunen M Aalberg V Comparison of the GHQ-36, the GHQ-12 and the SCL-90 as psychiatric screening instruments in the Finnish population Nord J Psychiatry 2003 57 233 238 10.1080/08039480310001418 12775300

[B17] Penninkilampi-Kerola V Miettunen J Ebeling HA Comparative assessment of the factor structures and psychometric properties of the GHQ-12 and the GHQ-20 based on the data from a Finnish population-based sample Scand J Psychol in press 10.1111/j.1467-9450.2006.00551.x 16987212

[B18] White HR Labouvie EW Towards the assessment of adolescent problem drinking J Stud Alcohol 1989 50 30 37 2927120 10.15288/jsa.1989.50.30

[B19] Aro H Life stress and psychosomatic symptoms among 14 to 16-year old Finnish adolescents Psychol Med 1987 17 191 201 3575572 10.1017/s0033291700013088

[B20] Koivumaa-Honkanen H Honkanen R Viinamaki H Heikkila K Kaprio J Koskenvuo M Life Satisfaction and suicide: a 20-year follow-up study Am J Psychiatry 2001 158 433 439 10.1176/appi.ajp.158.3.433 11229985

[B21] Koivumaa-Honkanen H Kaprio J Honkanen R Viinamäki H Koskenvuo M Life Satisfaction and depression in a 15-year follow-up of healthy adults Soc Psychiatry Psychiatr Epidemiol 2004 39 994 999 10.1007/s00127-004-0833-6 15583908

[B22] Williams RL A note on robust variance estimation for cluster-correlated data Biometrics 2000 56 645 646 10.1111/j.0006-341X.2000.00645.x 10877330

[B23] Stata, Version 8.0, College Station, TX http://www.stata.com

[B24] Bohne A Keuthen NJ Wilhelm S Deckersbach T Jenike MA Prevalence of symptoms of body dysmorphic disorder and its correlates: a cross-cultural comparison Psychosomatics 2002 43 486 490 10.1176/appi.psy.43.6.486 12444232

[B25] Mikkilä V Lahti-Koski M Pietinen P Virtanen SM Rimpelä M Associates of obesity and weight dissatisfaction among Finnish adolescents Public Health Nutr 2003 6 49 56 10.1079/PHN2002352 12581465

[B26] Reichborn-Kjennerud T Bulik CM Kendler KS Roysamb E Tambs K Torgersen S Harris JR Undue influence of weight on self-evaluation: a population-based twin study of gender differences Int J Eat Disord 2004 35 123 132 10.1002/eat.10252 14994348

[B27] Lindwall M Hassmen P The role of exercise and gender for physical self-perceptions and importance ratings in Swedish university students Scand J Med Sci Sports 2004 14 373 380 10.1046/j.1600-0838.2003.372.x 15546333

[B28] Rohrer JE Pierce JR Jr Blackburn C Lifestyle and mental health Prev Med 2005 40 438 443 10.1016/j.ypmed.2004.07.003 15530596

[B29] Scully D Kremer J Meade MM Graham R Dudgeon K Physical exercise and psychological wellbeing: a critical review Br J Sports Med 1998 32 111 120 9631216 10.1136/bjsm.32.2.111 PMC1756084

[B30] Thome J Espelage D Relations among exercise, coping, disordered eating, and psychological health among college students Eat Behav 2004 5 337 351 10.1016/j.eatbeh.2004.04.002 15488448

[B31] Mayville SB Williamson DA White MA Netemeyer RG Drab DL Development of the Muscle Appearance Satisfaction Scale: a self-report measure for the assessment of muscle dysmorphia symptoms Assessment 2002 9 351 360 10.1177/1073191102238156 12462755

[B32] Edward S Launder C Investigating Muscularity Concerns in Male Body Image: Development of the Swansea Muscularity Attitudes Questionnaire Int J Eat Disord 2000 28 120 124 10.1002/(SICI)1098-108X(200007)28:1<120::AID-EAT15>3.0.CO;2-H 10800022

[B33] Phillips KA Hollander E Rasmussen SA Aronowitz BR DeCaria C Goodman WK A severity rating scale for body dysmorphic disorder: development, reliability, and validity of a modified version of the Yale-Brown Obsessive Compulsive Scale Psychopharmacol Bull 1997 33 17 22 9133747

[B34] Sinisalo N Urheiluvalmisteiden epäpuhtaudet ja pakkausmerkintöjen lainmukaisuus [In Finnish: Sport supplements: impurities and regulatory compliance of package markings] Master's thesis 2004 University of Helsinki

[B35] Geyer H Parr MK Mareck U Reinhart U Schrader Y Schänzer W Analysis of non-hormonal nutritional supplements for anabolic-androgenic steroids – Results of an international study Int J Sports Med 2004 25 124 129 10.1055/s-2004-819955 14986195

[B36] Andrew T Hart DJ Snieder H de Lange M Spector TD MacGregor AJ Are twins and singletons comparable? A study of disease-related and lifestyle characteristics in adult women Twin Res 2004 4 464 477 10.1375/1369052012803 11780939

[B37] Hettema JM Neale MC Kendler KS A review and meta-analysis of the genetic epidemiology of anxiety disorders Am J Psychiatry 2001 158 1568 1578 10.1176/appi.ajp.158.10.1568 11578982

[B38] Kendler KS Walters EE Neale MC Kessler RC Heath AC Eaves LJ The structure of the genetic and environmental risk factors for six major psychiatric disorders in women. Phobia, generalized anxiety disorder, panic disorder, bulimia, major depression, and alcoholism Arch Gen Psychiatry 1995 52 374 383 7726718 10.1001/archpsyc.1995.03950170048007

[B39] Aarnio M Kujala UM Kaprio J Associations of health-related behaviours, school type and health status to physical activity patterns in 16 year old boys and girls Scand J Soc Med 1997 3 156 167 10.1177/140349489702500303 9360271

[B40] Moilanen I Rantakallio P Living habits and personality development of adolescent twins: a longitudinal follow-up study in a birth cohort from pregnancy to adolescence Acta Genet Med Gemellol (Roma) 1990 39 215 220 2239107 10.1017/s0001566000005444

[B41] Pietiläinen KH Kaprio J Rissanen A Winter T Rimpela A Viken RJ Rose RJ Distribution and heritability of BMI in Finnish adolescents aged 16 y and 17 y: a study of 4884 twins and 2509 singletons Int J Obes Relat Metab Disord 1999 23 107 115 10.1038/sj.ijo.0800767 10078843

